# Asenapine add‐on treatment for schizophrenia adults who received antipsychotics: A 52‐week, open‐label study

**DOI:** 10.1111/pcn.13540

**Published:** 2023-03-17

**Authors:** Taro Kishi, Yasuhiro Iwama, Yuji Sasagawa, Shuichi Hiraoka, Aya Kamei, Nakao Iwata

**Affiliations:** ^1^ Department of Psychiatry Fujita Health University School of Medicine Toyoake Japan; ^2^ Meiji Seika Pharma Co., Ltd. Tokyo Japan

Asenapine is used to treat schizophrenia and bipolar I disorder.^1^ Recent meta‐analyses demonstrated that asenapine is well‐tolerated and reduces overall symptoms in people with acute bipolar mania and schizophrenia.^2^, ^3^ Recently, a 52‐week open‐label, phase III extension study (NCT01244828 and JapicCTI‐132,325) of asenapine (5 or 10 mg BID) add‐on treatment for schizophrenia adults receiving antipsychotics was conducted.^4^ The study included participants with residual‐type schizophrenia (RTS),^5^ those who received antipsychotics in a polypharmacy setting, those who received high‐dose antipsychotics, those with treatment‐resistant schizophrenia, and those with geriatric schizophrenia. A total of 157 participants with a mean Positive and Negative Syndrome Scale^6^ total scores (PANSS‐T) of 90.20 ± 18.50 at a baseline were recruited.^4^ The change PANSS‐T from baseline to endpoint was −5.48 ± 13.34.^4^ Response rate was 18.3%.^4^ One of the five patient's death during the trial might be related to asenapine use (the detailed information: Supplementary text).^4^ Among the participants, 138 (87.9%) reported any adverse events.^4^ The number of individuals who discontinued due to adverse events was 37 (23.6%^4^).^4^ Nasopharyngitis was the most common adverse event, occurring in 21.0% of participants, followed by schizophrenia (13.4%), somnolence (12.7%), weight gain (12.7%), and oral hypoaesthesia (10.8%). The authors concluded that asenapine add‐on treatment was effective and safe for different types of schizophrenia patients.^4^ However, this study did not consider the disease severity at baseline (i.e. PANSS‐T) or age, resulting in heterogeneous characteristics and an unclear conclusion to identify the best responder to asenapine. Therefore, this *post‐hoc* analysis was performed in two groups [i.e. one group including individuals with treatment‐refractory schizophrenia (TRS), which included those with treatment‐resistant schizophrenia or individuals who received high‐dose antipsychotic and another group including individuals with RTS^5^] (the detailed information: Table S1).

The characteristics of the participants are presented in Table S2. The efficacy outcomes included the improvement of PANSS‐T, PANSS positive (PANSS‐P), and negative and general subscale scores. A paired *t*‐test was conducted to examine the differences in the efficacy scores between the baseline and endpoint. We also conducted a regression analysis to explore the relationship between PANSS scores and clinical variables such as sex, age, the severity of illness, use of lorazepam, use of antiparkinson drug, use of mood stabilizers, and Defined Daily Dose^7^ of antipsychotics at baseline (Table S3). This *post‐hoc* analysis included the last observation carried forward method in full analysis set population. The SAS software (SAS V9.4. SAS Institute Inc.) was used for all analyses.

The mean doses with a standard deviation of asenapine during the study in TRS and RTS groups were 12.30 ± 3.71 and 9.95 ± 1.66 mg, respectively. Asenapine add‐on treatment significantly improved the endpoint scores of all efficacy outcomes in both groups (Table 1).

**Table 1:** Changes in the PANSS score from baseline to endpoint

	Treatment‐refractory schizophrenia				Residual‐type schizophrenia			
	*n*	Mean	SD	*P*‐values*	*n*	Mean	SD	*P*‐values*
PANSS‐T								
Baseline score	79	94.62	15.45		53	83.28	20.97	
Endpoint score	79	88.68	19.51		53	78.11	23.70	
Change score	79	−5.94	14.88	**0.0007**	53	−5.17	12.18	**0.0032**
PANSS‐P								
Baseline score	79	22.06	5.48		53	17.09	6.55	
Endpoint score	79	20.39	6.20		53	15.64	6.67	
Change score	79	−1.67	4.64	**0.0020**	53	−1.45	4.07	**0.0121**
PANSS‐N								
Baseline score	79	25.54	5.59		53	25.30	6.27	
Endpoint score	79	24.29	6.29		53	23.26	7.46	
Change score	79	−1.25	3.62	**0.0029**	53	−2.04	4.49	**0.0017**
PANSS‐G								
Baseline score	79	47.01	8.28		53	40.89	10.89	
Endpoint score	79	44.00	10.39		53	39.21	12.25	
Change score	79	−3.01	8.57	**0.0025**	53	−1.68	5.11	**0.0204**

Abbreviation: PANSS‐(T, P, N, G): Positive and Negative Syndrome Scale (total score, positive subscale score, negative subscale score, general subscale score), SD, standard deviation.

* Bold indicates a statistically significant improvement of PANSS scores from baseline to endpoint.

The TRS subgroup that included individuals who had smoked in the previous 6 months (*n* = 25) had a greater reduction in PANSS‐T and PANSS‐P than the nonsmoker subgroup (Table S3). Smoking can induce CYP1A2 enzymes, lowering the expected plasma levels of certain antipsychotics.^8^ Therefore, asenapine add‐on treatment might improve schizophrenia symptoms in individuals who had smoked in the previous 6 months. The RTS subgroup that included individuals with initial PANSS‐P ≥ 20 (*n* = 17) had a more significant reduction in PANSS‐P at the endpoint than the subgroup that included individuals with initial PANSS‐P < 20 (Table S3). Other associations between some clinical factors and PANSS scores (Table S3) might not be robust because of the differences in patient numbers in each subgroup. However, discontinuation rates in both groups were similar (Table S4). Reported adverse events with a frequency of 10% or more in any groups included using an anticholinergic agent, oral paresthesia, somnolence, and weight gain (Table S5). The incidence of most adverse events in both groups was similar. However, the TRS group had 10% more individuals with weight gain than the RTS group. The recent meta‐analyses demonstrated that the risk of weight gain was not pronounced in asenapine compared with other antipsychotics such as olanzapine and zotepine.^2^, ^3^ Although we cannot classify participants by the type of antipsychotic medication they were taking other than asenapine, differences in concomitant antipsychotics might influence this result.

This post‐hoc analysis demonstrated that asenapine add‐on treatment might benefit individuals with TRS or RTS. Antipsychotic polypharmacy is frequent in clinical practice, despite the concern regarding the possibility of increased adverse effects and healthcare costs of antipsychotic polypharmacy.^9^ The physicians would need to keep an eye on those patients' health. As asenapine treatment reduced only about five points of PANSS‐T for both individuals, its clinical significance might be poor for some individuals who might be better off using clozapine.

## Disclosure statement

Mr. Iwama, Mr. Sasagawa, Dr. Hiraoka, and Ms. Kamei are full‐time employees of Meiji Seika Pharma Co., Ltd. The authors declare no conflicts of interest related to this study. Interests for the past 3 years are as follows: Dr. Kishi has received speaker's honoraria from Sumitomo, Eisai, Janssen, Kyowa, Otsuka, Meiji, Yoshitomo, Viatris, and Takeda, and research grants from Eisai, Japan Agency for Medical Research and Development, the Japanese Ministry of Health, Labor and Welfare, Grant‐in‐Aid for Scientific Research (C), and Fujita Health University School of Medicine. Dr. Iwata received speaker's honoraria from Sumitomo, Eli Lilly, Janssen, Eisai, Tanabe‐Mitsubishi, Otsuka, Meiji, Takeda, and Viatris as well as research grants from Eisai, Takeda, Sumitomo, and Otsuka.

## Supporting information


**Table S1.** The definition of individuals included in treatment‐refractory schizophrenia or residual‐type schizophrenia group.
**Table S2.** Demographic data of the participants.
**Table S3.** An association between changes in PANSS score and each clinical factor by regression analysis.
**Table S4.** Discontinuation rate.
**Table S5.** Adverse event.
