# Supplementary material for: Asenapine add‐on treatment for schizophrenia adults who received antipsychotics: A 52‐week, open‐label study
Source: Psychiatry Clin Neurosci. 2023 Mar 17;77(6):365–6. doi: 10.1111/pcn.13540 (PMC11488631; doi:10.1111/pcn.13540)
Supplement: Supplementary file 1 — Table S1. The definition of individuals included in treatment‐refractory schizophrenia or residual‐type schizophrenia group. Table S2. Demographic data of the participants. Table S3. An association between changes in PANSS score and each clinical factor by regression analysis. Table S4. Discontinuation rate. Table S5. Adverse event. [file PCN-77-365-s001.docx]

**Table S1. The definition of individuals included in treatment-refractory schizophrenia or residual-type schizophrenia group.**

| Individuals with schizophrenia who were residual-type | Individuals with residual-type schizophrenia (DSM-IV-TR) |
| --- | --- |
| Individuals with treatment-refractory schizophrenia | Individuals who met any of the following criteria   1. Individuals with schizophrenia received high dose antipsychotic (individuals treated with a dose greater than 18 mg/day haloperidol equivalent or 900 mg/day chlorpromazine equivalent for at least 1 month before baseline 2. Individuals with treatment resistant schizophrenia   (a) Treatment with at least two atypical antipsychotics at a dose equivalent to at least 12 mg/day haloperidol equivalent or 600 mg/day chlorpromazine equivalent for at least 4 weeks and no significant symptom reduction with any of these medications  (b) Taking clozapine within 12 weeks prior to baseline |

**Table S2. Demographic data of the participants.**

| Clinical factors | | Subgroups | Treatment-refractory schizophrenia  (Total n = 79) | | Residual-type schizophrenia  (Total n = 53) | |
| --- | --- | --- | --- | --- | --- | --- |
|  |  |  | n | % | n | % |
| Sex | | Male | 39 | 49.4% | 33 | 62.3% |
|  |  | Female | 40 | 50.6% | 20 | 37.7% |
| Age (median = 57*, y) | | 20-56 | 26 | 32.9% | 33 | 62.3% |
|  |  | ≥ 57 | 53 | 67.1% | 20 | 37.7% |
| Body weight (median = 57.8*, kg) | | < 57.8 | 46 | 58.2% | 28 | 52.8% |
|  |  | ≥ 57.8 | 33 | 41.8% | 25 | 47.2% |
| Body mass index (median = 22.5*) | | < 22.5 | 49 | 62.0% | 25 | 47.2% |
|  |  | ≥ 22.5 | 30 | 38.0% | 28 | 52.8% |
| Use of lorazepam | |  | 25 | 31.6% | 9 | 17.0% |
| Use of mood stabilizers** | |  | 27 | 34.2% | 12 | 22.6% |
| Use of antiparkinson drug | |  | 39 | 49.4% | 27 | 50.9% |
| Age of onset (median = 25*, y) | | < 25 | 39 | 49.4% | 23 | 43.4% |
|  |  | ≥ 25 | 40 | 50.6% | 30 | 56.6% |
| Duration of illness (median = 29*, y) | | < 29 | 29 | 36.7% | 34 | 64.2% |
|  |  | ≥ 29 | 50 | 63.3% | 19 | 35.8% |
| Duration of present episode | | unknown | 0 | 0.0% | 3 | 5.7% |
|  |  | < 2 months | 5 | 6.3% | 3 | 5.7% |
|  |  | ≥ 2 months | 74 | 93.7% | 47 | 88.7% |
| Number of episodes with inpatient | | unknown | 5 | 6.3% | 6 | 11.3% |
|  |  | < 4 | 23 | 29.1% | 30 | 56.6% |
|  |  | ≥ 4 | 51 | 64.6% | 17 | 32.1% |
| Past history of suicidal attempt | | unknown | 7 | 8.9% | 4 | 7.5% |
|  |  | Yes | 21 | 26.6% | 8 | 15.1% |
|  |  | No | 51 | 64.6% | 41 | 77.4% |
| Alcohol intake in past 6 months | | Yes | 5 | 6.3% | 5 | 9.4% |
|  |  | No | 74 | 93.7% | 48 | 90.6% |
| Smoking in past 6 months | | Yes | 25 | 31.6% | 14 | 26.4% |
|  |  | No | 54 | 68.4% | 39 | 73.6% |
| DDD of antipsychotics at baseline | | unknown | 21 | 26.6% | 4 | 7.5% |
|  |  | < 2.0 | 50 | 63.3% | 10 | 18.9% |
|  |  | ≥ 2.0 | 8 | 10.1% | 39 | 73.6% |
| PANSS scores | PANSS-T (median = 88*) | ≥ 88 | 51 | 64.6% | 17 | 32.1% |
|  | PANSS-P (median = 20*) | ≥ 20 | 55 | 69.6% | 17 | 32.1% |
|  | PANSS-N (median = 25*) | ≥ 25 | 43 | 54.4% | 27 | 50.9% |
|  | PANSS-G (median = 44*) | ≥ 44 | 52 | 65.8% | 18 | 34.0% |

*Median for AST population (n = 157)

**Mood stabilizers: valproate, lithium, carbamazepine, and/or lamotrigine

DDD: Defined Daily Dose, PANSS-(T, P, N, G): Positive and Negative Syndrome Scale (total score, positive subscale score, negative subscale score, general subscale score), y: years

**Table S3. An association between changes in PANSS score and each clinical factor by regression analysis.**

|  | **Treatment-refractory schizophrenia** | | | | **Residual-type schizophrenia** | | | |
| --- | --- | --- | --- | --- | --- | --- | --- | --- |
|  | P values** | | | | P values** | | | |
| Clinical factor | PANSS-T | PANSS-P | PANSS-N | PANSS-G | PANSS-T | PANSS-P | PANSS-N | PANSS-G |
| Sex | 0.7364 | 0.3154 | 0.2969 | 0.6303 | 0.7204 | 0.9969 | 0.6075 | 0.6850 |
| Age | 0.2468 | 0.4597 | 0.2752 | 0.2510 | 0.9928 | 0.6774 | 0.8639 | 0.6452 |
| Body weight | 0.6277 | 0.4310 | 0.8688 | 0.6272 | 0.6120 | 0.6733 | 0.2725 | 0.5580 |
| BMI | 0.6311 | 0.5816 | 0.8692 | 0.6415 | 0.1243 | 0.1326 | 0.3980 | 0.0850 |
| Use of lorazepam | 0.1375 | 0.1422 | 0.8079 | 0.0924 | 0.3643 | 0.3241 | 0.4519 | 0.4744 |
| Use of mood stabilizers | 0.6616 | 0.7656 | 0.7535 | 0.4644 | 0.9162 | 0.8466 | 0.9686 | 0.7110 |
| Use of antiparkinson drug | 0.2688 | 0.1586 | 0.2429 | 0.5070 | 0.7626 | 0.5385 | 0.7620 | 0.9721 |
| Age of onset>25* | 0.8284 | 0.8538 | 0.2897 | 0.8660 | 0.5456 | 0.2130 | 0.8615 | 0.7636 |
| Duration of illness >29 years* | 0.7540 | 0.1850 | 0.7328 | 0.7537 | 0.9672 | 0.6973 | 0.9357 | 0.7362 |
| Duration of present episode>2 months | 0.1607 | 0.0788 | 0.2159 | 0.3370 | **0.0070**^†^ | **0.0303**^†^ | **0.0266**^†^ | **0.0062**^†^ |
| Number of episodes with inpatient>4* | 0.2267 | 0.8850 | 0.0643 | 0.1603 | 0.6461 | 0.9975 | 0.6468 | 0.4925 |
| Past history of suicidal attempt | 0.9890 | 0.7575 | 0.8355 | 0.9561 | 0.9100 | 0.3938 | 0.8148 | 0.4433 |
| Alcohol intake in past 6 months | **0.0357^‡^** | **0.0390^‡^** | 0.0793 | 0.0757 | 0.9046 | 0.7554 | 0.8213 | 0.8134 |
| Smoking in past 6 months | **0.0493^§^** | **0.0019^§^** | 0.9646 | 0.0818 | 0.9522 | 0.4799 | 0.5162 | 0.8801 |
| DDD of antipsychotics at baseline>2* | 0.9987 | 0.7960 | 0.4451 | 0.6497 | 0.6873 | 0.6105 | 0.5214 | 0.9878 |
| PANSS-T>88* | 0.9280 | 0.8101 | 0.7518 | 0.8781 | 0.7367 | 0.3410 | 0.8282 | 0.8850 |
| PANSS-P>20* | 0.4884 | 0.1543 | 0.8894 | 0.7069 | 0.2800 | **0.0117^¶^** | 0.8140 | 0.4105 |
| PANSS-N>25* | 0.8601 | 0.6242 | 0.3486 | 0.7237 | 0.8866 | 0.8822 | 0.3642 | 0.7367 |
| PANSS-G>44* | 0.8461 | 0.9239 | 0.8372 | 0.6345 | 0.5559 | 0.8799 | 0.6245 | 0.3926 |

*The values were median.

**Bold indicates statistically significant factors.

†The subgroup that included patients with a current episode <2 months (n = 3) had a more substantial reduction in PANSS-T, PANSS-P, PANSS-N, and PANSS-G than the subgroup that included patients with a current episode >2 months.

‡The subgroup that included patients who consumed alcohol in the previous six months (n = 5) had a more significant reduction in PANSS-T and PANSS-P than the subgroup that included patients who did not drink alcohol in the previous six months.

§The subgroup that included patients who smoked in the last six months (n = 25) had a more significant reduction in PANSS-T and PANSS-P than those who had not smoked in the previous six months.

¶The subgroup that included patients with initial PANSS-P > 20 (n = 17) had a more significant reduction in PANSS-P at the endpoint than the subgroup that included patients with initial PANSS-P < 20.

BMI: body mass index, DDD: Defined Daily Dose, PANSS-(T, P, N, G): Positive and Negative Syndrome Scale (total score, positive subscale score, negative subscale score, general subscale score)

**Table S4. Discontinuation rate.**

|  | Treatment-refractory schizophrenia (n = 81) | | Residual-type schizophrenia  (n = 54) | |
| --- | --- | --- | --- | --- |
|  | n | % | n | % |
| All cause discontinuation | 40 | 49.4 | 25 | 46.3 |
| Discontinuation due to adverse event | 17 | 21.0 | 15 | 27.8 |
| Discontinuation due to inefficacy | 6 | 7.4 | 0 | 0.0 |
| Discontinuation due to withdrawal consent | 16 | 19.8 | 8 | 14.8 |
| Discontinuation due to lost follow up | 1 | 1.2 | 1 | 1.9 |
| Discontinuation due to other reasons | 0 | 0 | 1 | 1.9 |

**Table S5. Adverse event.**

|  | Treatment refractory schizophrenia  (n = 81) | | Residual type schizophrenia  (n = 54) | |
| --- | --- | --- | --- | --- |
|  | n | % | n | % |
| Use of anticholinergic agents | 38 | 48.1 | 27 | 50.9 |
| Extrapyramidal symptoms | 5 | 6.2 | 3 | 5.6 |
| Oral paraesthesia | 9 | 11.1 | 8 | 14.8 |
| Hypersalivation | 3 | 3.7 | 2 | 3.7 |
| Somnolence | 7 | 8.6 | 9 | 16.7 |
| Insomnia | 5 | 6.2 | 1 | 1.9 |
| Fall | 4 | 4.9 | 2 | 3.7 |
| Headache | 4 | 4.9 | 3 | 5.6 |
| Constipation | 5 | 6.2 | 3 | 5.6 |
| Nausea | 4 | 4.9 | 4 | 7.4 |
| Hyperprolactinemia | 2 | 2.5 | 4 | 7.4 |
| Weight gain | 13 | 16.0 | 3 | 5.6 |

**Supplementary text**

The information for one patient who died due to caused asenapine use.

This patient (70 years, male) was classified in residual-type schizophrenia group in this *post-hoc* analysis. When the physician visited the patient on the 19^th^ day starting the asenapine add-on trial, the patient told the physician that there were not any side effects. However, when a family visited on his house at the night of the next day (20^th^ day), the patient died with sitting in a chair. The principal Investigator determined that the cause of his death might relate to use of the investigational drug (i.e., asenapine).

**Author contribution statement**

Dr. Kishi developed the study concept and design. Mr. Iwama, Mr. Sasagawa, Mr. Hiraoka, and Ms. Kamei had full access to all data and takes full responsibility for the integrity of the data and the accuracy of the data analysis. All authors acquired and interpreted the data, and wrote the manuscript. Dr. Iwata supervised the review.
